# Volatile Profile of Raw Lamb Meat Stored at 4 ± 1 °C: The Potential of Specific Aldehyde Ratios as Indicators of Lamb Meat Quality

**DOI:** 10.3390/foods7030040

**Published:** 2018-03-16

**Authors:** Ioannis Konstantinos Karabagias

**Affiliations:** Laboratory of Food Chemistry, Department of Chemistry, University of Ioannina, 45110 Ioannina, Greece; ikaraba@cc.uoi.gr; Tel.: +30-697-828-6866

**Keywords:** lamb meat, freshness, volatile compounds, aldehydes, aldehyde ratios, quality control

## Abstract

The objectives of the present study were: (a) to evaluate the aroma evolution of raw lamb packaged in multi-layer coating film and stored at 4 ± 1 °C, with respect to storage time and (b) to investigate whether specific aldehyde ratios could serve as markers of lamb meat freshness and degree of oxidation. Volatile compounds were determined using headspace solid phase microextraction coupled to gas chromatography/mass spectrometry. Results showed that the most dominant volatiles were 2,2,4,6,6-pentamethyl-heptane, hexanal, 1-octen-3-ol, 1-hexanol, carbon disulfide and *p*-cymene. Volatile compound content was increased during storage time. However, statistically significant differences were recorded only for hexanal, heptanal, and nonanal (*p* < 0.05). Additionally, the evolution of aldehydes during storage recorded a positive Pearson’s correlation (*r*) (*p* < 0.05), whereas hexanal to nonanal, heptanal to nonanal, octanal to nonanal ratios, along with the sum of aldehydes to nonanal ratio, were positively correlated (*r* = 0.83–1.00) with the degree of oxidation (mg malonic dialdehyde per kg of lamb meat). A perfect Pearson’s correlation (*r* = 1) was obtained for the ratio hexanal to nonanal. Therefore, this ratio is proposed as an indicator of lamb meat freshness and overall quality.

## 1. Introduction

Colour and flavour are the most important quality attributes consumers use to evaluate meat quality, and thus, acceptability and preference. Raw meat has little odour and only a mild serum-like taste, which is described as salty, metallic and “bloody” with a sweet aroma [1]. 

Sheep meat is a good source of polyunsaturated (linoleic (C18:2, *n*-6) and α-linolenic (C18:3, *n*-3)) and monounsaturated (oleic acid (C18:1 *n*-9)) fatty acids which have beneficial effects for human health. On the other hand, sheep meat contains significant amounts of saturated fatty acids with a prospective negative impact on human health, when consumed daily in high amounts [2].

Among different factors which influence the meat flavour, animal diet, especially its lipid content, is the most important, as the main source of volatile compounds [3]. The lipid fraction of meat, especially phospholipids, undergoes autoxidation, producing a large number of volatile compounds such as acids, aldehydes, ketones and alcohols and promoting the formation of other components such as nitrogen and sulphur-containing compounds [3]. In addition, lamb age, gender and weight may also affect the volatile profile of lamb meat [2]. What should not be forgotten is that biological processes such as rigor-mortis or post-mortem glycolytic fluxes might modify the volatile fraction of fresh meat [4].

The characteristic flavour of fresh meat, fermented meat products or dry cured ham has been reported to be a subtle balance between non-volatile compounds with taste properties and volatile compounds which interact with each other along with proteins and lipids [1,5].

Many methods have been developed to enhance the efficiency of the isolation of the volatiles in foods, such as vacuum distillation, simultaneous distillation and extraction, purge and trap or headspace techniques. Solid-phase microextraction (SPME) is an inexpensive, easy, rapid and efficient technique as compared to others [6,7,8,9,10].

With respect to volatile compound analysis, gas chromatography coupled to mass spectrometry (GC-MS) is the technique that is most generally used. The literature involving research on volatile compounds in raw meat generally considers beef, duck, pork and poultry meat. However, very few publications deal with the volatile fraction originating from refrigerated storage and the large majority of the works deal with meat products and cooked meat using emerging technologies [1,7,11,12,13]. Surprisingly, there are only a few studies that have been carried out on the determination of volatile compounds in raw meat by using mild temperature analytical procedures [1,11,12,13,14,15] and/or the use of specific volatile compounds for the differentiation of meat origin [13]. In addition, the specific sense of smell that products of animal origin possess may provide useful information regarding the analysis of quality control [16,17].

Lamb meat is an important source of monetary profit for meat producers, exporters, local butcher’s shops and supermarkets in the region of Epirus and holds a special position in the food chain and casualties of local people.

Based on the aforementioned, the aims of the present study were: (a) to evaluate the volatile profile of raw lamb meat during storage under refrigeration and (b) to investigate whether specific aldehyde ratios could be correlated with shelf life and degree of oxidation test data [15], serving thus as markers of lamb meat freshness and overall quality.

## 2. Materials and Methods

### 2.1. Lamb Meat Samples, Packaging and Analysis Conditions

Leg of lamb meat (hind shank) samples in chunks, of dimensions ca. 2 × 2 × 2 cm, were provided from different chilled carcasses, 24 h post slaughter, by a local meat processing company (SVEKI S.A., Rodotopi, Ioannina, Greece). Lambs belonged to the Fries–Arta breed, which is a cross breed with high-yielding animals, farmed intensively in the lowlands. Lambs were 3–6 months old, male, with a slaughter weight of 12–13 kg. To avoid any source of contamination, lamb meat samples were transported to the laboratory in insulated polystyrene boxes on ice within 1 h of the chopping process. Prepared samples consisted of 150 ± 10 g in weight, were immediately placed in low density polyethylene/polyamide/low density polyethylene (LDPE/PA/LDPE) barrier pouches (6–7 chunks per pouch), 75 μm in thickness, having an oxygen permeability of 52.2 cm^3^ m^−2^ day^−1^ atm^−1^, at 75% relative humidity (RH), 23 °C and a water vapour permeability of 2.4 g m^−2^ day^−1^ at 100% RH, 23 °C. The experimental procedure was carried out on 36 samples: 2 bags × 2 replicates × 9 sampling days. Samples were stored at −18 °C in order to avoid any source of deterioration and prepared daily prior to headspace solid phase microextraction coupled to gas chromatography/mass spectrometry analysis (HS-SPME-GC/MS).

### 2.2. Determination of Lipid Oxidation

The degree of lipid oxidation in lamb meat samples was estimated using thiobarbituric acid assay (TBA) according to the methodology described in a previous work [18]. The value of TBA shows the degree of oxidation of the fats of a product. Measuring the degree of oxidation of polyunsaturated fatty acids is an indicator of the stability of a food’s fat in oxidation. In addition, the value of TBA shows the content of malondialdehyde (MDA) (mg/kg) in a fatty foodstuff since the latter compound originates from lipid peroxidation of unsaturated fatty acids. During the reaction, two molecules of TBA and one molecule of MDA react to give a red-coloured product which may be determined spectrophotometrically at 532 nm [18]. Absorbance measurements were accomplished using a UV-VIS spectrophotometer (PerkinElmer, Lambda 25, East Lyne, CT, USA).

### 2.3. Determination of Volatile Compounds

A divinyl benzene/carboxen/polydimethylsiloxane (DVB/CAR/PDMS) fiber 50/30 μm (Supelco, Bellefonte, PA, USA) was used to extract headspace volatile compounds from raw lamb meat. Prior to use, the fiber was conditioned following the manufacturer’s recommendations. Approximately, 2 g of lamb meat from different parts of the lamb legs were placed in a 20 mL crimp-cap vial (72 × 20 mm) equipped with PTFE/silicone septa. Then, 10 μL of an internal standard (4-methyl-2-pentanone, of initial concentration C = 640 ng/mL, Sigma Aldrich (Darmstadt, Germany)) was added at the walls of the vial, and the vial was wrapped. It was vortexed for 5 min in order for the internal standard to be spread homogenously at the surface of raw lamb meat. The vial was then maintained at 50 °C in a water bath under stirring at 600 rpm during the entire extraction procedure, which was 30 min (15 min was the equilibration time and 15 min was the adsorption time). It should be stressed that when the heating of the raw meat product is somehow “mild” during analysis, the volatile profile can be considered as pure information, originating from raw meat. Volatile compounds analysis was carried out on day 1, day 5, and day 9 of storage. Each sample was run in duplicate (*n* = 2).

### 2.4. GC/MS Instrumentation and Method Conditions

A Hewlett-Packard, model 6890, gas chromatograph coupled to a Hewlett-Packard, model 5973, mass spectrometric detector (Agilent Technologies, Wilmington, DE, USA) was used for the determination of lamb meat volatile compounds. A HP-INNOWAX (polyethylene glycol capillary column, Agilent, Santa Clara, CA, USA) (30 m × 320 μm i.d., × 0.50 μm film thickness) was used, with helium as the carrier gas (purity 99.999%) at 1.4 mL/min flow rate. The injector and MS-transfer line were maintained both at 250 °C, respectively. For the analysis of lamb meat volatile compounds, oven temperature was held at 40 °C for 5 min, increased to 110 °C at 5 °C/min, and finally further increased to 240 °C at 8 °C/min (5 min hold). The fiber was maintained in the injector for 10 min. Electron impact mass spectra were recorded at 29–300 mass range with 3 scans/s. An electron ionization system was used with ionization energy of 70 eV. Regarding the detector, the temperature of MS quadrapole was held at 150 °C and that of MS source at 230 °C, respectively. Finally, a split ratio 2:1 was used.

### 2.5. Mass Spectral Data Processing

The identification of compounds was achieved by comparing the mass spectra of the chromatographic peaks with those of the Wiley 275 MS database. For semi-quantification, compound concentration was expressed as ng/g based on the ratio: peak area of analyte/peak area of internal standard (4-methyl-2-pentanone), considering the density (0.8 g/mL) of the internal standard. For validation purposes, volatile compounds having only ≥80% similarity with the Wiley mass spectral library were tentatively identified using GC-MS spectra. For the determination of linear retention indices, a mixture of n-alkanes (C_6_–C_20_) dissolved in n-hexane was employed. The mixture was supplied by Sigma Aldrich (Darmstadt, Germany). The calculation was carried out for components eluting between n-octane and n-eicosane using Equation (1).

### 2.6. Formatting of Mathematical Components

Kovats indices were calculated using Equation (1):

(1) $$I=100\times \left(n+\left(\frac{tr\left(unkown\right)-tr\left(n\right)}{tr\left(N\right)-tr\left(n\right)}\right)\right)$$

where *I =* {*\displaystyle I=*}, *I* = Kovats retention index; *n =* {*\displaystyle n=*}, *n* = the number of carbon atoms in the smaller n-alkane; *N =* {*\displaystyle N=*}, *N* = the number of carbon atoms in the larger n-alkane; *tr =* {*\displaystyle t_*{*r*}*=*}, *tr* = the retention time.

Equation (1) is the main source for the calculation of Kovats index values, since the temperature was programmed.

### 2.7. Statistical Analysis

The evolution of each volatile compound with respect to storage time was estimated using paired samples *t*-test analysis at the confidence level *p* ≤ 0.05. Correlations were obtained by Pearson’s bivariate correlation coefficient (*r*), at the confidence level *p* < 0.01. All statistical treatments were accomplished using the SPSS version 20.0 statistics (IBM, Armonk, NY, USA) software.

## 3. Results

**Figure 1:**
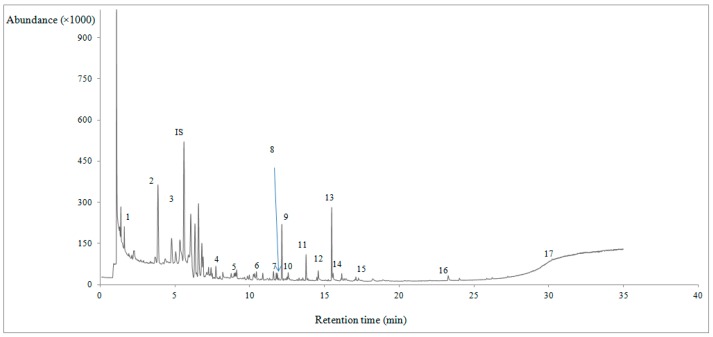
A typical gas chromatogram of raw lamb meat on day 5 of storage. Volatile compounds are numbered according to retention time given in Table 1. 1: carbon disulfide, 2: 2,2,4,6,6-pentamethyl-heptane, 3: toluene, 4: hexanal, 5: *o*-xylene, 6: heptanal, 7: 1-pentanol, 8: 3-octanone, 9: *p*-cymene, 10: octanal, 11: 1-hexanol, 12 :nonanal, 13: 1-octen-3-ol, 14: 1-heptanol, 15: 1-octanol, 16: benzothiazole, 17: 15-crown ether. IS: internal standard.

Significant variations were observed in the volatile fraction of raw lamb meat during storage under refrigeration, including hexanal, heptanal, and nonanal, as specified by paired samples *t-*test (*p* ≤ 0.05). There was observed an increasing trend in all volatile compounds with respect to storage time, with the exception of octanal, toluene, and carbon disulfide, where fluctuations were recorded (Table 1). A typical gas chromatogram, pointing out the volatile compounds identified in the headspace of raw lamb meat is given in Figure 1.

Considerable variations in the volatile profile of raw unprocessed meat have been reported previously in numerous studies, involving the volatile profile of the raw meat of duck, goose, pork [1], beef [14,17] and lamb [2,15,19]. What is worth mentioning is that the collective results of breed comparisons suggested that genetic effects on lamb flavour were minor as compared with the effects of other factors such as age, sex, feeding/diet, oxidation, lipid content, myoglobin and pH [20]. In these factors mentioned previously in the literature, the role of air packaging technology, in terms of real market analysis, in the development of raw lamb’s meat aroma during storage should be also investigated. In real market analysis, consumers often purchase fresh, unprocessed meat from supermarkets or butcher’s shops, packaged or covered with paper roll, and store it under refrigeration prior to cooking, roasting or grilling. In that sense, the development of aroma in packaged lamb meat during storage at 4 ± 1 °C has not been yet investigated. The specific trend in lamb meat aroma evolution, along with the characteristics/role of each class compound determined in the present study, follows the text sequence.

### 3.1. Alcohols

Garcia et al. [21] and Barbieri et al. [22] indicated that 1-pentanol and 1-hexanol, were produced from the degradation of homologous aldehydes during lipid and amino acid oxidation. 1-Hexanol has a herbal and fatty odour, whereas 1-pentanol has a pleasant, sweet or fruity odour [20]. Respective odour thresholds are 2500 and 10,000 ng/g [23].

1-Octen-3-ol has been reported to be a common product of oxidation. Several precursors have been reported for 1-octen-3-ol, including linoleic and arachidonic acids [24]. In a recent study, 1-octen-3-ol was found at higher amounts in the meat of lambs fed with olive cake as compared to those fed with linseed [2]. It is characterized by a mushroom-like, grassy odour [20]. Its odour threshold has been reported to be 1 ng/g [23].

In accordance with other researchers, the alcohols identified in the present study have been previously reported in studies involving fresh meat of pork, duck, goose [1], beef [14,17], turkey [11], cattle [13] and lamb [2,15], respectively. We may then conclude that these alcohols are typical volatile markers of raw and unprocessed meat.

#### 3.1.1. Aldehydes

Aldehydes, in general, are the main representatives of volatile compounds derived from ruminant animal’s meat [25]. According to Mottram [26], the straight chain aldehydes are compounds derived from the oxidation of fat. Hexanal, 2-heptenal, and 2,4-decadienal, (derived from linoleic acid), were associated with a diet rich in small amounts of plants [27,28]. Aldehydes act as important components in the transmission of certain flavors. The short-chain aldehydes tend to have a pungent or acidic odour, with the increasing of fatty acid chain length, and the degree of unsaturation.

Among aldehydes that are secondary products of fat oxidation [29], hexanal is a product resulting from the oxidation of unsaturated omega-6 fatty acids, having a low odour detection limit of 5.87 ng/g [30] and a paint-like, herbal, and rancid taste [31]. Heptanal has been reported to have a fatty odour, whereas octanal and nonanal a soapy or waxy odour [20,32]. The most abundant aldehydes identified in raw lamb meat were hexanal, followed by nonanal, heptanal, and octanal.

It should also be mentioned that the aforementioned aldehydes have been reported previously, adding to the volatile profile of lamb meat packaged under aerobic conditions [2], in agreement with present results.

#### 3.1.2. Ketones

Ketones possess a low detection odour threshold [14]. The only ketone that was identified in raw lamb meat was 3-octanone. This volatile compound has been associated with the animal breed or the type of animal feeding background [13,14]. In addition, 3-octanone was found to be favorable to thermal processing of raw, trimmed of fat, cattle meat [13]. It is characterized by a fruity odour and an odour threshold ranging between 16–28 ng/g [33].

#### 3.1.3. Hydrocarbons: Aliphatic and Benzene Derivatives

The hydrocarbons identified (aliphatic and aromatic) may originate from the animal food origin. These compounds are absorbed in fat tissue [34], as they are obtained from the degradation of lipids [21]. The aliphatic hydrocarbon 2,2,4,6,6-pentamethyl-heptane has been reported previously, aiding to the volatile profile of raw cattle meat [13,14] and lamb meat [2] having a characteristic odour [35].

On the other hand, there is a class of aromatic hydrocarbons that is characterized by a high degree of unsaturation and unusual stability. The most common member of this class is benzene (C_6_H_6_). When one of the positions on the ring has been substituted with another atom or group of atoms, the resulting compound is a mono-substituted benzene. The more common ones are toluene, *o*-xylene, and *p*-cymene. In the present study, these benzene derivatives contributed to the volatile pattern of raw lamb meat, recording a high rate of evolution, especially toluene and *p*-cymene, during storage (Table 1). This is also in agreement with the work of Insausti et al. [14]. However, their overall variation during storage was insignificant (*p* > 0.05) (Table 1). Some researchers have reported that aromatic hydrocarbons may be formed during the thermal decomposition of hydrocarbons, fats, and proteins [36]. However, in the present study these volatile compounds were found in raw, not thermally treated lamb meat. This is in agreement with the results of Insausti et al. [14], Saraiva et al. [17] and King et al. [37].

The presence/and or the combination of such mono-substituted benzenes, may play an important role in the overall flavour development of raw lamb meat, even though no individual compound of this group has a characteristic meat-like odour [38]. This is also in agreement with the results of Min et al. [39]. The typical odour of toluene and *o*-xylene is sweet, pungent or paint-like [38,40]. Toluene has an odour threshold of 2900 ng/g whereas that of *o*-xylene ranges between 620–1000 ng/g [40].

Insausti et al. [14] and Vasta et al. [13,15] documented the contribution of such compounds to the overall aroma development of raw beef, cattle and lamb meat, respectively.

#### 3.1.4. Sulfur Compounds

Generally, sulfur compounds arise from sulfur-containing amino acids, cysteine and methionine, produced by proteolytic enzymes found in psychrotrophic bacteria (usually present in slaughterhouses). In the present study, carbon disulfide and benzothiazole were the only sulfur compounds determined. Carbon disulfide (CS_2_) may also be derived from dithio-carbamate fungicides used in agriculture [41]. Carbon disulfide has a pleasant, sweet or ether-like odour [42] and an odour threshold ranging between 1.6–420 ng/g [23]. In the study of Saraiva et al. [17] carbon disulfide contributed to the overall aroma of raw beef packaged under vacuum or modified atmosphere packaging during refrigerated storage.

On the other hand, benzothiazole, is an aromatic heterocyclic compound found commonly in nature [43] and has an odour threshold of 80 ng/g [23]. It has been reported to have an unpleasant, dirty, metallic or pyridine-like odour [44]. The thiazole derivatives have been identified among the volatile components of coffee, boiled meat, boiled potatoes, sterilized milk and beer. Thiazoles and thiazolines are mainly formed by Strecker degradation of sulfur amino acids (particularly cysteine). Lorenz et al. [45] found that sulfur compounds were the main components of meat extract. Various studies indicated that sulfur compounds were present at higher amounts in animals which grazed normally, as compared to animals fed with concentrated food. In that sense, Young et al. [28] indicated that dimethyl-sulfide and dimethyl-sulfone were detected in lamb fat and were associated with pasture activities. The sulfur compounds in meat arise during cooking by the reaction of hydrogen sulfide (formed by cleavage of the amino acid cysteine) with carbonyl compounds derived from the Maillard reaction [26,46]. Given that lamb meat was not thermally treated, carbon disulfide and benzothiazole could be considered as typical volatiles of raw lamb meat associated with pasture diets/activities.

#### 3.1.5. Ethers

Vapors of certain ethers may be used as insecticides, miticides, and fumigants for soil [47]. The only ether that was identified in the volatile fraction of raw lamb meat was 15-crown ether, recording insignificant variations (*p* > 0.05) with respect to storage time. Its presence in the volatile pattern of unprocessed lamb meat may be attributed to environmental contamination.

## 4. Discussion

The volatile fraction of raw lamb meat was largely dominated by aldehydes, which are secondary products of oxidation and have been considered as markers of fat oxidation during storage time [48]. However, several volatiles that are not directly related to lamb meat flavour were also identified, namely benzothiazole, *o*-xylene, toluene, and crown ether-15. Such compounds may be considered as artifacts originating from environmental or external contamination and may not be considered as “natural” or unambiguous meat volatiles.

The evolution of aldehydes with respect to storage time recorded positive Pearson’s correlation (*r*) (*p* < 0.05). Respective *r* values for the groups hexanal–nonanal, heptanal–nonanal, and octanal–nonanal were 0.82, 0.44, and 0.72, through the 9 days of storage. However, the groups hexanal–nonanal and octanal–nonanal recorded excellent *r* values equal to 1 for the first 5 days of storage, in which raw lamb meat retained a fresh character based on microbiological (total viable counts < 7 log CFU/g) and sensory data (odour and taste) [18]. This was not the case for the group heptanal–nonanal, in which a perfect negative Pearson’s correlation was recorded (*r* = −1). The evolution of the aforementioned aldehydes through the 9 days of storage is given in Table 2.

Specific aldehyde ratios, namely hexanal to nonanal (Hex/Non), heptanal to nonanal (Hept/Non), octanal to nonanal (Oct/Non), and sum of hexanal, heptanal, and octanal to nonanal ratio (Hex + Hept + Oct/Non) (Table 2) recorded strong positive Pearson’s correlation with the degree of oxidation values (TBA test) expressed as mg malonic dialdehyde per kg of lamb meat (mg MDA/kg) throughout storage (1.4 mg MDA/kg on day 1, 2.8 mg MDA/kg on day 5, and 3.8 MDA/kg on day 9) [18]. Respective Pearson’s correlation values were *r* = 1.00, *r* = 0.87, *r* = 0.83, and *r* = 0.89 (Table 2). It should be stressed that the hexanal to nonanal ratio has been proposed as an indicator of the level of oxidation in olive oil [49], whereas variations in hexanal content with respect to storage time could serve as indicators of flavour deterioration in cooked pork [48]. In addition, octanal and nonanal (among other volatiles) have been proposed as indicators of raw beef quality stored under refrigeration [17].

Based on the aforementioned, the ratios Hex/Non, Oct/Non, and Hex + Hept + Oct/Non may be proposed as indicators of freshness and degree of oxidation, enhancing the quality of fresh lamb meat stored under refrigeration.

## 5. Conclusions

Results of the present study showed that the aroma of raw, unprocessed lamb meat is the outcome of the combination of specific volatile compounds. Storage time proved to be a parameter that significantly affected the evolution of hexanal, heptanal, and nonanal (*p* < 0.05). Furthermore, the evolution of hexanal, heptanal, octanal, and nonanal during storage under refrigeration, along with the Hex/Non, Oct/Non, and Hex + Hept + Oct/Non ratios could be proposed as overall indicators of lamb meat freshness and degree of oxidation, since they were strongly correlated with shelf life and TBA test data.

Special attention should be given to hexanal to nonanal ratio, which could serve as a unique marker of freshness and overall quality, since was perfectly correlated with the results of degree of oxidation (TBA test). Therefore, this ratio value—equal or lower than 2.45—is proposed as an indicator of lamb meat freshness and overall quality.

As an executive summary, the present study, unique in the literature, aids the fast quality control of raw lamb meat that enters the market by proposing specific aldehydes or aldehyde ratios as markers of raw lamb meat quality, in support of some previous studies on other food products [17,48,49].

## Figures and Tables

**Table 1:** Volatile compounds (average ± SD, ng/g) determined in raw lamb meat under air packaging with respect to storage time (days).

VOCs	RT	KI	Day 1	Day 5	Day 9	MSLM (%)	MI	*p*
			Content (ng/g)	Content (ng/g)	Content (ng/g)			
*Alcohols*								
1-Pentanol	11.77	1233	11.77 ± 0.01	24.73 ± 0.01	56.77 ± 7.83	83	MS/KI	*p* = 0.144
1-Hexanol	13.77	1342	27.70 ± 0.01	71.29 ± 47.72	236.92 ± 19.62	83	MS/KI	*p* = 0.224
1-Octen-3-ol	15.49	1459	96.45 ± 0.01	145.40 ± 156.77	397.11 ± 25.70	90	MS/KI	*p* = 0.151
1-Heptanol	15.59	1461	11.98 ± 0.01	37.40 ± 4.91	63.20 ± 8.69	86	MS/KI	*p* = 0.121
1-Octanol	17.29	1557	23.55 ± 0.02	30.90 ± 0.01	57.27 ± 16.65	91	MS/KI	*p* = 0.057
*Aldehydes*								
Hexanal	7.75	1105	163.20 ± 0.01	231.02 ± 92.34	340.37 ± 165.89	96	MS/KI	*p* = 0.040
Heptanal	10.34	1185	36.64 ± 0.02	29.38 ± 0.76	60.94 ± 6.75	98	MS/KI	*p* = 0.043
Octanal	12.60	1299	21.02 ± 0.01	25.83 ± 26.67	56.59 ± 23.91	91	MS/KI	*p* = 0.083
Nonanal	14.61	1393	80.98 ± 0.04	94.84 ± 1.20	97.52 ± 56.52	91	MS/KI	*p* = 0.001
*Ketones*								
3-Octanone	11.87	1266	23.44 ± 0.02	36.39 ± 46.46	66.98 ± 31.91	95	MS/KI	*p* = 0.073
*Heterocyclic*								
Benzothiazole	23.27	1954	17.24 ± 0.02	24.53 ± 0.66	45.52 ± 0.30	91	MS/KI	*p* = 0.062
*Benzene derivatives*								
Toluene	6.57	1027	381.95 ± 0.06	221.99 ± 51.56	735.19 ± 84.40	91	MS/KI	*p* = 0.099
*o*-Xylene	9.13	1164	25.02 ± 0.10	27.24 ± 30.36	72.86 ± 9.48	95	MS/KI	*p* = 0.114
*p*-Cymene	12.18	1280	29.35 ± 0.07	103.31 ± 15.33	181.68 ± 62.54	95	MS/KI	*p* = 0.139
*Hydrocarbons*								
2,2,4,6,6-pentamethyl-heptane	3.86	<800	171.27 ± 26.42	226.12 ± 360.83	435.97 ± 196.77	83	MS	*p* = 0.074
*Sulfur compounds*								
Carbon disulfide	1.62	<800	825.58 ± 359.41	125.31 ± 44.37	376.24 ± 44.34	90	MS	*p* = 0.168
*Ethers*								
15-Crown ether	30.23	2432	5.11 ± 0.01	5.77 ± 2.82	20.41 ± 14.99	90	MS/KI	*p* = 0.158

VOCs: volatile compounds. RT: retention time. SD: standard deviation. Every value is the average of two determinations (*n* = 2). MSLM: mass spectral library match. MI: method of identification, with reference to mass spectra (MS) provided by Wiley 275 MS database and Kovats indices (KI). *p*: significance as assessed by paired samples *t*-test, at the confidence level ≤0.05.

**Table 2:** Aldehyde ratios and malonic dialdehyde development with respect to storage time (days).

Aldehyde Ratio-MDA	Day 1	Day 5	Day 9	Pearson’s (*r*)
Hexanal–Nonanal	2.02	2.44	3.49	1.00
Heptanal–Nonanal	0.45	0.31	0.62	0.87
Octanal–Nonanal	0.26	0.27	0.58	0.83
Sum of (Hexanal, Heptanal, Octanal)–Nonanal	2.73	3.02	4.70	0.89
MDA (mg/kg)	1.4	2.8	3.8	

Aldehyde ratios were obtained by dividing average values of each aldehyde presented in Table 1. MDA: malonic dialdehyde. Pearson’s correlation coefficient *r* was considered at the confidence level *p* < 0.05.
